# Using Owner Return as a Reinforcer to Operantly Treat Separation-Related Problem Behavior in Dogs

**DOI:** 10.3390/ani10071110

**Published:** 2020-06-29

**Authors:** Erica N. Feuerbacher, Kristy L. Muir

**Affiliations:** 1 Virginia Tech, Blacksburg, VA 24061, USA; 2 Lilly Hill Farm, Lakeland, FL 33810, USA; kmuir@lilyhillfl.com

**Keywords:** separation-related problem behavior, domestic dog, reinforcement, behavioral treatment, operant conditioning

## Abstract

**Simple Summary:**

Separation-related problem behavior in dogs is a challenging behavioral issue, often manifesting as destructive behavior or excessive vocalization when the dog is left alone. More knowledge is needed on effective treatments. In this study, we explored whether we could train dogs to engage in desirable behavior over increasing durations of owner absence by rewarding the dog with the owner returning. After collecting baseline data, dogs started treatment. When the dogs showed no problem behavior or a specific desirable behavior, the owner returned as a reward for the good behavior. We gradually increased the time the dog was left alone based on their observed performance in earlier trials. We demonstrated that dogs’ ability to stay alone without problem behavior increased compared to baseline. Nevertheless, after four sessions, only one dog was able to stay alone for at least 5 min, demonstrating how challenging this behavioral issue is to treat and how much time might be involved in improving behavior. While our research helps fill the many gaps in this field, it also points to the need for more research to further increase treatment efficacy.

**Abstract:**

Separation-related problem behavior (SRPB) is a severe behavioral issue in which dogs engage in a variety of undesirable behaviors when the owner is absent, such as destructive behavior and excessive vocalization. Given the severity and high prevalence of SRPB, finding effective treatments is crucial. To date, most treatments have relied on habituation to increase tolerance to owner absence. Additionally, research has typically not utilized direct observations of the dog’s behavior and the treatment implemented with unknown treatment integrity. We evaluated an operant approach to SRPB using owner return as the reinforcer. After collecting baseline, we enrolled five dogs for treatment. Treatment involved differential reinforcement of either absence of problem behavior or occurrence of specific desirable behaviors. Behavioral criteria for delivering reinforcement changed based on the dog’s performance assessed through direct observation. We coached owners to ensure treatment integrity on each trial. From baseline, mean time to SRPB was 27.1 s. During treatment, all dogs increased their ability to stay alone without SRPB compared to baseline, indicating that contingent owner return can be a useful treatment. However, despite four training sessions, only one dog was able to stay alone for over 5 min. Our data demonstrate the slow-going progression of this SPRB treatment and the challenges of this behavioral issue.

## 1. Introduction

Separation-related problem behavior (SRPB) is an umbrella term for behavioral issues in dogs when left alone or separated from an owner or other person with whom the dog has had an extensive history of reinforcement. Common problem behaviors reported as SRPB are excessive barking or whining, excessive salivation, property destruction, and possible concomitant injury to the dog, escape or escape attempts, and improper elimination [1,2]. The term separation-related problem behavior encompasses both the more specific, clinical diagnosis of separation anxiety, as well as other instances of problem behavior such as destruction due to frustration or boredom [3]. 

SRPB is a severe behavioral disorder; after aggression, it is the most frequently reported behavioral problem of dogs referred to a behavioral clinic [4], with 35% of dogs referred to a professional for behavioral issues exhibiting SRPB [1]. In a survey of adopters of rescued greyhounds, SRPB was the top behavioral issue that adopters were “very concerned” about (35.7% of adopters) [5]. Problematic behaviors are also one of the most common reasons for surrendering a dog (34.2%) [6], and one of the most common behavioral issues reported was destructive tendencies.

Along with being a severe behavioral issue, it is also a common one. A survey of dog owners walking in southern Hampshire, England found that 29% of dogs currently exhibited SRPB and an additional 21% had previously [7]. In post-adoption follow-ups, between 14% and 18% of dogs adopted from a New York shelter [8] were reported to have separation anxiety, 38% of dogs adopted from a shelter in England exhibited SRPB [9], and nearly 43% of rescued greyhounds exhibited SRPB [5]. While high rates in shelter dogs might not be surprising, by 18 months of age, over 50% of border collies and Labrador retrievers that had been reared by breeders, and did not pass through a shelter, had displayed SRPB [7]. Other research [10,11] further supports that this is not an issue restricted to shelter dogs, as it was once speculated SRPB was a result of the dog’s loss of an attachment figure when surrendered [12]. 

### 1.1. Treatments and Treatment Efficacy

Given the prevalence and severity of SRPB, finding effective treatments is essential. Much research on SRPB has focused on pharmacological interventions, for example clomipramine [13,14], fluoxetine [15,16], and diazepam [15]. Typically, these studies included a behavioral treatment plan for owners to carry out during the study. The behavior plans usually had three main stages and between 7 and 12 specific, bullet-pointed instructions for owners to follow within those three stages. However, research into owner compliance shows that compliance dropped when owners were given more than five instructions and that they were most likely to comply with easy steps (e.g., not punishing the dog for separation anxiety behaviors) but not with more complex and potentially behaviorally relevant steps, such as desensitization and uncoupling departure cues [4]. Thus, treatment integrity is likely low in those studies, nor was it objectively assessed. 

A second issue for most studies on SRPB is the mode in which treatment efficacy was assessed, which typically relies on owner reports. Owners report globally on the dog’s behavior over a several-week time span [13,14,15]. Not only did these time points rely on owners to accurately remember the dog’s behavior for the time since the last report, people are known to report improvements due to treatment simply because they have invested in the treatment, regardless of its actual efficacy (see [17] for a discussion of the 26 different psychological reasons clients will report improvement without any actual improvement, or improvement but not due to treatment). Thus, these owner reports, especially the global measures, are very subjective and prone to influences other than treatment efficacy. 

Finally, it is worth noting that many of the components in the behavioral treatment plans provided as adjunctive treatment in the pharmacological studies on SRPB are not evidence-based interventions [13,14] and, in fact, tend to address SRPB as a relationship issue (e.g., not allowing the dog to sleep in the owner’s bed or bedroom, not giving attention if the dog initiates interaction). However, etiological research has found no connection between “spoiling” a dog (e.g., allowing the dog to sleep on the bed) and whether the dog exhibits SRPB [2,10], so those interventions are unlikely to bring about meaningful behavior change. 

Very few studies exist on the efficacy of behavioral treatments themselves for SRPB. One of the few that evaluated a behavioral treatment (without the confounds of also testing a pharmacological agent) for SRPB used a combination of counterconditioning and systematic desensitization [18]. This research improved upon the studies referenced under the pharmacological studies by relying on an evidence-based behavioral plan (systematic desensitization), which was first used to treat phobias in humans [19]. Treatment efficacy assessment was also more objective as owners rated the severity of SRPB (based on observable evidence in the house—destruction, rearrangement of household items) after each departure, rather than asking for a report several days or weeks after many of these departures occurred, and by having an independent observer (a person familiar with the dog but not the owner) also rate the severity of behavioral evidence as a corroboration of the owner’s rating [18]. These methodological tactics were a significant improvement in assessing objective treatment efficacy for SRPB. 

In the systematic desensitization component of this study [18], owners were told to leave the dogs for 5 min and then return, regardless of what the dog was doing upon return; if the dog showed no signs of problem behavior (PB), owners could increase the length of absence by an additional 5 min but had to return to the last duration of no occurrence of PB if the dog showed signs of separation distress. If the owners returned and there was evidence of PB, they were told to ignore the dog for 30 min. However, because owners did not monitor their dog’s behavior and only looked for evidence of destruction, they could not deliver their consequences (ignoring for 30 min) if the dog had emitted problem vocalization.

The initial criterion of leaving the dog for 5 min [18] was likely too long for most dogs. A video analysis of the behavior of those engaged in SRPB showed that most SRPB occurred within 10 min of owner departure, with the mean latency to vocalization being 3.25 min and the mean latency to destruction 7.13 min [20]. In the treatment study [18], how owners proceeded (the rate of duration increase) was unmoderated and might have accounted for differential outcomes in treatment efficacy. Indeed, by the owner’s ratings and our visual assessments from the presented data, dogs varied in the percentage of trials in which no PB was evident (range of 36–93% successful trials). Interestingly, the dog that had the highest percentage of successful trials also had the owner who increased the departure duration the slowest and had the greatest number of short departures. The authors concluded that systematic desensitization was the critical component for successful treatment and that counterconditioning and other behavioral advice did not influence the behavioral outcome. 

### 1.2. Operant Approaches for Behavioral Treatment

Desensitization is a common component of SRPB treatment plans [1,21] and consists of the owner departing for successively longer periods of time. The owner’s return is not contingent on the dog’s behavior—the owner simply determines how long they think the dog can tolerate being alone and return when that time elapses. Systematic desensitization involves exposing the animal to multiple instances of brief departures. After a set amount of time elapses, the owner returns, regardless of dog behavior. Across trials, the duration of owner departure increases. 

However, it is possible that operant processes can be at work in this procedure, although not explicitly identified, and that the systematic desensitization procedure might actually rely on operant conditioning. Prior research has demonstrated that owner access is a reinforcer for dogs [22], suggesting that any behavior the dog is engaged in when the owner returns could be reinforced. Thus, in the systematic desensitization process, if the dog was not engaging in PB when the owner returned, this would inadvertently reinforce more desirable behaviors. However, because the operant, response-dependent contingency was not made explicit in the systematic desensitization process, there could also be instances in which the dog was engaged in PB when the owner returned, and this behavior would be strengthened, thereby delaying treatment progress. If this procedure were made more explicitly operant (i.e., owner return is *contingent* on desirable behavior) the treatment could potentially produce more stable behavioral improvement. 

A treatment of aggression through an explicitly operant procedure (shaping a calm, alternative behavior) was faster (hours to effective treatment) than typical desensitization and counterconditioning treatments (weeks or months to effective treatment) and utilized the natural reinforcer that was maintaining the undesirable behavior to shape and maintain the new response [23]. Other research has further established the utility of using the reinforcer maintaining the problem behavior to establish and maintain desirable behavior during treatment, e.g., [24,25,26]. Given the prior research that suggests owner access can function as a reinforcer for dogs [22], we hypothesized that owner return could function as a reinforcer to shape and maintain desirable behavior in dogs with SRPB. 

### 1.3. Current Study

We recruited six dogs with SRPB (five completed the study) and investigated using owner return as a reinforcer for desirable behavior using differential reinforcement schedules [27]. We collected baseline data of the dog’s behavior during a 30-min owner absence and calculated latency to PB and duration of time spent in different behaviors, following the behavioral categories of prior video analyses of dogs with SRPB [20]. Subsequently, dogs participated in four treatment sessions that lasted 30–60 min per session. In the treatment sessions, desirable behavior during owner absence was consequated with the owner returning, sitting, and interacting normally with the dog for 1 min. 

We initially started all dogs in a differential reinforcement of other behavior (DRO) schedule of reinforcement in which the owner returned contingently on the absence of problem behavior. We chose to start all dogs in a DRO to maximize likelihood that the dog would contact reinforcement compared to specifying a specific behavior the dog had to engage in. In a DRO schedule, the criteria for reinforcer delivery is the nonoccurrence of the target behavior for a specified period of time [27,28,29], such that there is no specified behavior that the animal has to engage in as long as it does not engage in the PB. For example, compulsive circling in a dog was treated by using a DRO with owner attention being provided for the absence of circling [26].

For two dogs, while implementing the DRO, specific desirable behavior emerged such that we transferred these dogs to a differential reinforcement of alternative behavior (DRA) schedule, in which the dog had to engage in a specific behavior or set of behaviors to access the owner return. DRA schedules specify what behavior or set of behaviors the animal must engage in for reinforcement to be delivered [27,28,29], rather than any behavior except the PB, as in a DRO. While the behaviors chosen for a DRA are typically topographically dissimilar from the target PB, they are not necessarily physically incompatible with the target PB. For example, an olive baboon that engaged in self-injury (biting her arms and legs and pulling her hair to the point of leaving bald patches) was successfully taught to engage in lip-smacking as an alternative behavior to self-injury [24] by having keepers give attention contingent on lip-smacking. 

DRO and DRA schedules both have advantages and disadvantages (see [28] for a full review of the two procedures). DRO schedules are easy to implement and can allow the learner to contact reinforcement regularly for a wide range of behaviors. However, because the DRO does not specify what behavior the animal should engage in, it is possible that the animal does not engage in the target PB (and reinforcement delivered), but the behavior the animal engaged in, while not the target PB, is also undesirable. DRA schedules solve this in that the behavior that produces reinforcement is specified. However, the DRA might also take longer to reduce the occurrence of the undesirable behavior because the DRA requires the acquisition of the specified desirable response.

We increased criteria for dogs on DRO and DRA across trials as long as the dog did not exhibit PB and decreased if the dog did exhibit PB in a given trial. We measured trial-by-trial progress of the dogs across the four treatment sessions, recording occurrence or absence of PB, time left alone, and occurrence of desirable behavior (DRA only). Our study is the first to detail trial-by-trial objective reports of SRPB behavior while undergoing treatment, rather than relying on owner reports or ratings. Thus, we were able to measure latency to PB in each trial, the exact percentage of successful trials (including trials involving only vocalizations which leave no permanent products to evaluate), the probability that if PB occurred on a given trial, it would occur on the subsequent trial, and the highest criteria each dog reached without exhibiting PB, which we compared to their baseline data.

## 2. Baseline

All research (Baseline and Treatment) was undertaken following approval from the Institutional Animal Care and Use Committee of the University of Florida, where the first author was enrolled during data collection; IACUC number 201307875.

### 2.1. Methods

We recruited five dogs from the community, all of which had been reported by their owners to regularly engage in SRPB (see Table 1 for dog demographics). All dogs had lived with their owners for at least 4 months. A sixth dog that had been formally diagnosed with separation anxiety by a veterinary behaviorist and took 50 mg of fluoxetine daily exhibited no SRPB in Baseline, despite owner reports, so it was not included in the study. 

We collected baseline data on each dog’s SRPB. Each owner was provided a webcam that they placed in a discreet location in the room the dog typically stayed when the owner left, and where the owner would like the dog to be able to stay without PB during owner absences. During one of the owners’ routine departures from the house, they recorded the dog’s behavior on the webcam. For Harrison, Scarlet, and Sadie, the treatment area was the main living areas of the house (entryways, living room, kitchen). For Mei and Tristan, the treatment area was their crate. Both dogs could stay calmly in their crates as long as the owner did not show any departure behaviors or was not absent. We asked each owner to leave for at least 30 min. From these Baseline sessions, we determined the topography of problem behavior that the dog typically engaged in (e.g., excessive vocalization, destruction), as well as the duration and frequency of the problem behavior. We also measured the latency from owner departure to initiation of problem behavior. 

### 2.2. Analysis

All sessions were video-recorded, and we scored the baseline video sessions for the behaviors in accordance with prior research [20] (Table 2). We coded each video two times: once for locomotion behaviors and once for vocal behaviors as those two large classes of behavior could occur simultaneously (e.g., the dog was circling and whining). We coded Harrison and Sadie a third time for the behavior of panting and Mei a third time for lip licking as the frequency of these behaviors made it challenging to code with the other behavior categories and would have jeopardized the integrity of our coding. We calculated duration dogs spent engaged in each behavior, except for lip licks, which were calculated as a frequency. We also calculated latency until undesirable behavior occurred. We calculated interobserver agreement (IOA) for 20% of the baseline videos by comparing the behavior coded and the time at which it was coded from two independent observers. If each observer coded the same behavior within 3 s of each other, that was counted as an agreement. If either the behavior coded or the time at which it was coded did not meet those criteria, it was counted as a disagreement. The IOA was 88.7%.

### 2.3. Results and Discussion

Owners for four of the five dogs (Harrison, Sadie, Scarlet, and Tristan) recorded their dogs’ behavior for the requested 30 min. However, for Mei, the owner terminated the session early (12.5 min) due to excessive vocalization that the owner monitored via webcam. Given that prior research reported that most PB occurred within the first 10 min after owner departure [20], and this session was terminated for PB; we decided that this was an acceptable Baseline session. 

**Figure 1:**
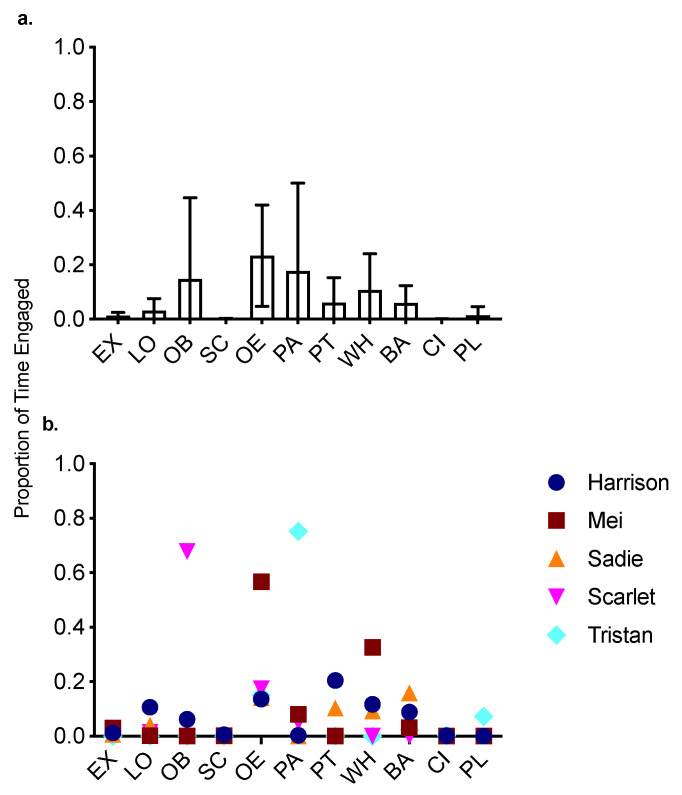
(**a**). Mean and SD of the proportion of time dogs engaged in various behavioral categories during Baseline. (**b**)**.** Individual dogs’ proportion of time engaged in various behavioral categories. *EX*—exploration, *LO*—locomotion, *OB*—oral behavior, *SC*—scratching, *OE*—oriented to environment, *PA*—passive behavior, *PT*—panting, *WH*—whining, *BA—*barking, *CI*—circling, *PL*—playing.

Figure 1a shows the mean and standard deviations of the proportion of Baseline session time that the five dogs included in Treatment engaged in certain behaviors. Only the behaviors that we observed dogs engage in are shown. Figure 1b shows the individual dogs’ proportion of time engaged in those same behaviors. On average, dogs spent the highest proportion of time oriented to the environment (23.4%). Except for Mei (56.7%), the other four dogs engaged in behavior oriented to the environment between 13.6% (Harrison) and 17.3% (Scarlet) of the time. The higher percentage of time for Mei might be an artifact of her shorter baseline session; it is possible that with a longer session, this behavior would have decreased over time, thus producing a lower percentage in a longer session. The next most frequently observed behavior on average was passive behavior (17.7%), with dogs ranging from 0–75.2% of time passive.

Combining whining and barking into the category *vocalization,* dogs spent on average 16.7% of their time vocalizing. Within dogs, whining ranged from 0% of the time (Scarlet and Tristan) to 32.6% (Mei). Barking ranged from 0% of the time (Scarlet) to 15.9% (Sadie). 

Combining oral behavior and scratching into the category *destruction,* dogs spent on average 14.9% of their time engaged in destruction. The high percentage of time engaged in destruction was mainly driven by Scarlet, who engaged in oral behavior for nearly 70% of the 30 min baseline session (Figure 1b). Harrison also engaged in oral behavior for 6.7% of the session. Only two dogs exhibited scratching (Harrison and Mei). 

Panting was observed 6.1% of the time on average; two dogs exhibited panting (Harrison and Sadie) but engaged in it for a substantial amount of time (20.4% and 10.3%, respectively). Circling was rarely observed (0.1% of the time). We observed lip licking in one dog (Mei) (37 lip licks in baseline duration = 2.96 lip licks/min).

Our video analysis was based on prior research on the behaviors of dogs exhibiting SRPB when left alone [20]. Although that prior study and our overarching research questions were different, and thus they had a much larger sample size (*n* = 23), comparing our results with theirs can speak to the generality of their and our findings. Taking into account the potentially large behavioral differences between individuals and the potential for our data set (*n* = 5) to be affected by a single outlier, our results in general are similar to those in that study [20]. Typically, the dogs in both studies spent proportionally the most time orienting to the environment (ours: 23.4%; theirs: 21%), engaging in vocalization (ours: 16.8%; theirs: 22.95%), and passive behavior (ours: 17.7%; theirs: 12%). However, the percentage of time engaged in destruction in our study (14%) was higher than that seen in the prior work (6%) [20], and the dogs in our study exhibited more oral behavior and less scratching than the dogs in their study (oral behavior: 14.8%, 1%, respectively; scratching: 5%, 0.1%, respectively). 

**Figure 2:**
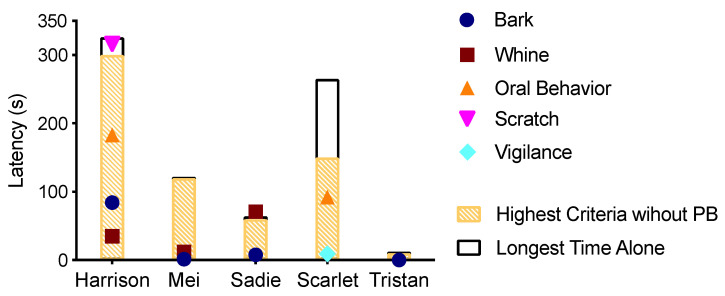
Highest criteria reached (hatched bars) and longest amount of time alone without PB (open bars) during Treatment for each dog. Symbols indicate latency to specific PB for each dog as observed in Baseline.

We also measured latency to problem behavior (PB) in Baseline sessions. Table 3 and Figure 2 show the latency to any PB in the Baseline session. We included Vigilance on this for Scarlet (running to the side window and jumping up on the sill with her front feet); although it is not a problem behavior per se, it was undesirable in that it was incompatible with a relaxed state, and, in the Treatment phase, we did not deliver reinforcement if she was engaged in that behavior. For all dogs, the first instance of PB occurred within 2 min (mean latency to first PB 27.1 s). For Tristan, the latency was listed as 0 s as he began barking before his owner left the room. Our results are similar to prior video analysis results [20] in that those researchers found that most PB occurs within 10 min of owner departure (mean latency to vocalization 3.25 min and mean latency to destruction 7.13 min). However, the dogs in our study showed even shorter latencies to PB. Given the short latencies to PB, we suspect that the dogs in our study could be diagnosed with separation anxiety or frustration-based SRPB, and that their SRPB was likely not due to boredom, which could be potentially managed through environmental enrichment rather than requiring operant or Pavlovian conditioning. As such, all five that showed PB were enrolled in our treatment. The behaviors observed in Baseline were used to determine the PB topographies that we would target for reduction in the Treatment phase (see Table 4 for specific behaviors). 

## 3. Treatment 

### 3.1. Methods

The five dogs that exhibited PB in Baseline were included in the Treatment phase. Treatment took place in the same setting where we measured Baseline for each dog. We set up a webcam connected to a computer to monitor the dog’s behavior during treatment. The webcam was oriented so that owner departure and return through the door could be seen, as well as providing a maximal view of the experimental space to monitor the dog’s behavior. The computer was placed outside the front door, and the experimenter monitored the dog on the screen during owner absence. The owner(s) remained near the experimenter so that they could re-enter the house quickly when the experimenter indicated that the dog had met the behavioral requirement. Each dog participated in four treatment sessions, each approximately 30–60 min long. Sessions were scheduled in accordance with owner availability, such that not all sessions were conducted on consecutive days, but sessions were scheduled not more than 1 week apart. 

#### 3.1.1. Contingencies 

Owners chose a novel verbal cue that they would say to the dog, such as “stay there,” before they started their departure. This was a cue that they did not currently use with the dog but that was easy for them to remember to say. We included this cue with hopes that it would function as a discriminative stimulus that predicted owner return contingent on non-SRPB behavior. Owners were instructed to not use this cue outside of training until training was complete. This cue was intended to help the dog discriminate between the training sessions, in which desirable behaviors would result in owner return, and non-training owner departures outside of the treatment sessions, in which those same calm behaviors would not necessarily result in owner access. We hoped that this discrimination would prevent long owner absences that occurred between training sessions from impeding training. Additionally, if the cue gained stimulus control over appropriate behavior, it could then be used whenever the owner left and would control the dog’s appropriate behavior during the separation.

For all trials, the experimenter monitored the dog’s behavior via webcam so that she could prompt the owners when to return to the dog on the current trial and could determine the correct criteria for the next trial. Contingent on the dog meeting behavioral criteria for a given trial (see below for criteria), owners re-entered the room with the dog and remained there for 1 min. Owners were instructed to sit down and engage normally with the dog during the 1 min owner return. If the dog showed problem behavior on a trial, the owner waited until 5 s had elapsed without problem behavior before returning and engaging normally with the dog, as they did on trials with no PB. We included the additional 5 s delay after problem behavior ended to avoid inadvertently reinforcing undesirable behavior [30]. For Mei, Sadie, and Tristan, there was only one owner for the dog. For Harrison and Scarlet, there were two owners, who both left and returned together during treatment. For simplicity, we will refer to *owner* in the following sections and this will refer to either the individual owners or pairs of owners. 

#### 3.1.2. Trials and Criteria

Trials began when the owner(s) gave the novel verbal cue and then proceeded to depart. After they gave the verbal cue, they exited the house for the specified amount of time. When the dog met the specified behavioral criteria, the owner(s) returned for 1 min. After 1 min, the owner(s) again gave the verbal cue and exited the room for the next trial. 

##### First Trial

For the first trial for all dogs, the initial criterion for reinforcement was the owner exiting, closing the door, and, returning immediately (0 s delay), as long as there was no problem behavior (denoted as DRO + 0 s). Three dogs (Harrison, Mei, and Scarlet) were successful in this trial and proceeded along the criteria hierarchy as indicated in Table 5 and detailed below. Two dogs (Sadie and Tristan) engaged in problem behavior before the owner could exit and close the door. Thus, for these two dogs, we determined where in the departure sequence they showed problem behavior and established their initial criteria to be below that, to increase the likelihood that the dog would successfully meet the behavioral criteria and contact reinforcement. 

##### Subsequent Trials

Across trials, we increased criteria for reinforcement. After two successful trials (i.e., no PB exhibited), we increased the criteria as prescribed (Table 5). For the DRO schedule, the length of time the dog had to not engage in PB to access reinforcement increased by 5 s intervals up to 15 s, at which point the criteria increased by 15 s intervals up to 60 s, after which the intervals increased by 30 s. 

If a dog exhibited PB in a trial, we dropped to the last successful criteria. For example, if the dog showed undesirable behavior at DRO + 15 s, the criteria for the next trial would be DRO + 10 s, and the dog would have to complete two successful trials (no PB) at these criteria before increasing back to DRO + 15 s. However, if the dog showed problem behavior at the easier criteria (e.g., DRO + 10 s), the dog would further drop back to the next easiest level (e.g., DRO + 5 s). 

For the two dogs that exhibited PB on the initial trial in Session 1 (Sadie and Tristan), their initial criteria were arranged to include steps prior to the owner exiting (stepping across the door threshold). The initial training criteria at which Sadie and Tristan were successful are detailed in Table 6. Reinforcement for all of the trials for Sadie and Tristan was provided as for all other trials (owner sitting down and interacting with the dog for 1 min). As Tristan was in a crate and Sadie’s owner stepped over a small baby gate, behind which Sadie remained, on the way to the door, both were able to effectively step away from the dog while still inside the house and return contingent on desirable behavior. Once the owner could exit the room and close the door behind them, the sessions proceeded as for the other dogs. 

As indicated in Table 6, the initial schedule of reinforcement for all dogs was a differential reinforcement of other behavior (DRO) schedule, such that the owner returned as long as the dog had not engaged in problem behavior for the requisite duration. Three dogs (Mei, Sadie, and Tristan) remained in the DRO condition throughout the treatment sessions. 

##### Differential Reinforcement of Alternative Behavior (DRA)

For the other two dogs (Harrison and Scarlet), while the DRO was in effect, we observed specific desirable behaviors (e.g., head turn, or sitting/lying down) emitted by the dog. Given that these were specific desirable behaviors, all of which were suggestive of a more relaxed state of the dog and not as engaged in locating the owner (i.e., not staring at the door through which the owner exited), we decided to reinforce these behaviors specifically. Thus, we moved these dogs to a DRA schedule in which owner return was contingent on the dog emitting the specific targeted response for each dog (see Table 4 for a list of target behaviors). On Trial 4, Harrison emitted a head turn so we had the owner immediately return when we observed the head turn. Following that, Harrison continued on a DRA for head-turning. On Trial 8 (Harrison) and Trial 31 (Scarlet) each dog lay down, so we had the owner immediately return contingent on when we observed the dog lie down. Following those trials, each dog continued on a DRA for sitting or lying down (for Harrison, we discontinued the DRA for head turns). After two successful trials in which the owners immediately returned contingent on the target DRA behavior (turning head, sitting, or lying down), we added a duration component to the DRA (Table 5) with the same time increments that we used for the DRO schedule. For example, after Harrison successfully lay down or sat on two trials and the owner returning immediately contingent on him lying down or sitting, the next criteria for Harrison would have been lying down or sitting for 5 s. 

For the first trial of each subsequent session, we relaxed the criteria from the last successful criteria of the previous session to increase the likelihood that the dog would meet the behavioral criteria and contact reinforcement, while still ensuring the dog made further progress across sessions. If the dog engaged in PB on the first trial, we reduced the criteria on the next trial to a level that, based on the latency to PB in Trial 1, the dog would be successful (i.e., a shorter duration than the latency to PB). For Session 4 for Sadie, after she exhibited PB on the two initial trials of the session before the owner exited the house, we dropped criteria back to levels at which the owner remained in the house. The initial and final criteria for each session for each are listed in Table 6.

After the four treatment sessions were complete, we provided owners with a written description of the procedures so they could continue training. 

### 3.2. Analysis 

The experimenter live-coded whether problem behavior occurred in a given trial to decide what the criteria should be for the upcoming trial. Additionally, we video-recorded all sessions and used the videos to code when the owner left, when the door closed and the dog lost visual access to the owner, when the owner returned, if and when the dog engaged in PB, and if and when the dog engaged in targeted desirable behavior (for those dogs that were in a DRA). We calculated the percentage of trials that were successful (the dog emitted no PB) and the total time the dog was alone (as this could vary from the criteria if the dog was on a DRA). For dogs on a DRA schedule of reinforcement, we also quantified the latency to the desirable alternative behavior across trials. Finally, for all dogs we calculated the conditional probability that, if problem behavior occurred on one trial, problem behavior would occur on a subsequent trial, to determine whether inadvertently pushing a dog over its threshold resulted in a continued backslide in treatment. 

An independent observer double-coded 20% of Treatment videos to calculate IOA by comparing the behavior or event (owner exit/return) coded and the time at which it was coded from two independent observers. If each observer coded the same behavior or event within 3 s of each other, that was counted as an agreement. If either the behavior/event coded or the time at which it was coded did not meet those criteria, it was counted as a disagreement. The IOA ranged from 86.7% to 100% with a mean of 95.1%.

### 3.3. Results and Discussion

We treated all five dogs using owner return as a reinforcer. Table 4 details the specific behaviors we targeted for reduction for each dog, and Table 3 reports the number of trials each dog participated in across the four sessions. We successfully increased all dogs’ ability to stay home alone without exhibiting problem behavior compared to their behavior in Baseline (Figure 2 and Table 3). Using one-tailed nonparametric Wilcoxon matched-pairs signed-rank test, we found the highest criteria reached was significantly greater than the latency to PB (time dog could spend alone without PB in baseline) (*W* = 15, *p* < 0.05). 

Because dogs in the DRA did not necessarily immediately emit the behavior targeted in the DRA and a specific duration of the target behavior was required for reinforcement (Table 5), the time the dogs were actually alone was often longer than indicated by the DRA requirement. For example, at one treatment level, Harrison was required to sit or lie down for 5 min for the owner to return; the actual time alone was 5 min 26 s because he lay down 26 s after the owner left, and then remained there for the 5 min until the criteria was met and the owner returned (See Figure 2 and Table 3 for actual time alone during the longest successful trials for dogs that treated using a DRA). The longest time dogs successfully spent alone was also significantly greater than the latency to PB (time dog could spend alone without PB) in baseline (*W* = 15, *p* < 0.05). Additionally, by the end of treatment, the length of time the dogs successfully spent alone was longer than each of the dogs’ latency to problem behavior (except whining in Sadie) such that we could have reasonably expected all of the problem behaviors to have been exhibited within that interval of time. 

**Figure 3:**
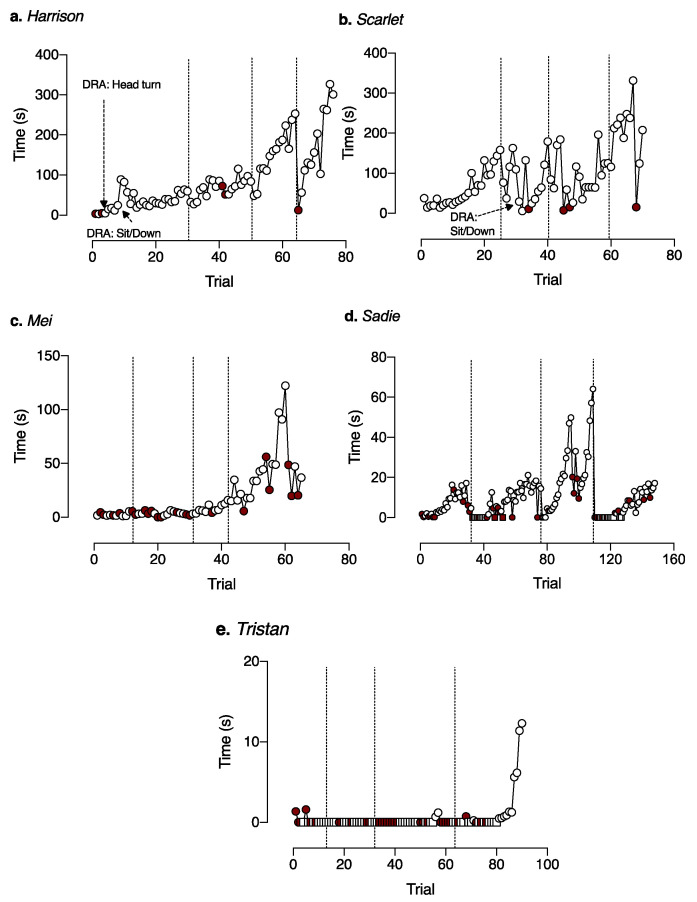
Trial-by-trial data for each dog (**a.** Harrison; **b.** Scarlet; **c.** Mei; **d.** Sadie; **e.** Tristan) during Treatment. On each trial, we plotted either the time the dog was alone (for successful trials with no PB; open symbols) or the latency to PB on unsuccessful trials (filled symbols). Circle symbols indicate that the criteria were such that the owner was exiting the house on that trial. Square symbols indicate criteria were such that the owner was staying inside the house on that trial. For all trials in which the owner remained in the house, time dog was alone are recorded as 0 s. Latency to any PB that occurred while the owner was still in the house was recorded as 0 s. Thus, open circles with latency 0 s indicate the owner successfully exited the house without the dog exhibiting PB (DRO + 0 s), whereas open squares indicate a successful trial in which the owner did not exit the house. Time alone on successful trials might exceed the DRO or DRA time criteria if (1) the dog was engaged in vigilant behavior on a DRO and we waited for vigilant behavior to end before enforcing the DRO contingency (vigilant behavior was not reinforced but was not considered PB), or (2) the dog did not engage in the target desirable behavior for some time on a DRA. Dotted vertical lines indicate the end of one session and the start of a new session.

Despite the improvement, the duration of owner absence that we reached was still fairly short (Table 3). The dogs that reached the highest criteria were Harrison and Scarlet. These results align with the percentage of successful trials (Table 3 and Figure 3), in which Harrison and Scarlet also had the highest percentages of successful trials (96.1% and 94.3%, respectively). Figure 3, which plots the time each dog was alone on successful trials and the time to PB on trials on unsuccessful trials, illustrates the slow-going nature of treating SRPB. While all dogs demonstrated progress within and between sessions, progress was slow and prone to setbacks (Table 6 delineates the beginning and ending criteria across sessions). For Sadie and Tristan, many of the trials were conducted at criteria in which the owner did not exit (step across the threshold; Figure 3d,e). Additionally, despite Mei and Sadie making progress within trials (Figure 3c,d), they were often unsuccessful on the first trial of each session, such that the criteria reset to very low levels on the second trial and there was less across-session progress.

As noted earlier, our sessions were not on contiguous days, and the owners left their dogs alone on intervening days. We did not ask owners to train outside of our treatment sessions because we wanted to monitor behavior change within an experimental setting and ensure treatment integrity. Despite using a unique cue which we hoped would serve as a discriminative stimulus for the DRO or DRA being in effect, the results from Sadie indicate that it might not be sufficient, and preventing the dog from being left alone between treatment sessions will likely maximize the speed and success of training. Based on our results, we recommend future research to minimize those absences and video record any absences that do occur for inclusion in the treatment analysis. Still, the qualitative observations of prior studies match our quantitative measurements regarding the treatment of SRPB: it is “unwieldy, demanding, and especially tedious” [1] (p. 244).

The relative success of Harrison and Scarlet, both in the relatively high criteria reached and the high percentage of successful trials, warrants further discussion. Harrison and Scarlet were differentiated from the other dogs by a few factors: the main behavior reported as concerning by owners was destructive problem behavior rather than excessive vocalization, although Harrison would also bark and whine, whereas the main behavior reported as concerning by owners for Mei, Sadie, and Tristan was excessive vocalization. It is possible that treating vocalization PB is much more difficult than treating destructive PB, although the latter might seem more severe to an owner. Dogs vocalize under many situations, and barking or whining might be a very strong response for these dogs such that, even when we change the current contingency for barking, it is very hard to extinguish or modify. Anecdotally, even when Sadie was quiet during owner absence, once the owner opened the door and was walking back, Sadie often barked and the continued owner presence and attention might reinforce barking as a general behavioral class, making modification during absence more difficult.

A second differentiating factor for Harrison and Scarlet was that they were able to move to a DRA rather than a DRO. It is possible that the fact they were able to exhibit specific desirable behaviors (lying down/sitting) indicated that they were not as aroused as the other dogs and more likely for any behavioral treatment to be successful. It is also possible that because dogs that engage in destruction typically had longer latencies to problem behavior, we had more opportunity to identify specific, desirable alternative behavior to treat with a DRA. 

Finally, the difference of being on a DRA and a DRO could itself be a factor. A DRA might be a more effective treatment than a DRO. A DRA establishes a contingency between a specific behavior or behaviors to access reinforcement, whereas a DRO does not have such a specific behavior-reinforcer contingency—the animal can do anything *except* the response targeted for reduction to access reinforcement. Although studies with humans have demonstrated the utility of using a DRO [31], the speed of acquisition of desirable responses using a DRO compared to a DRA has not been evaluated. 

**Figure 4:**
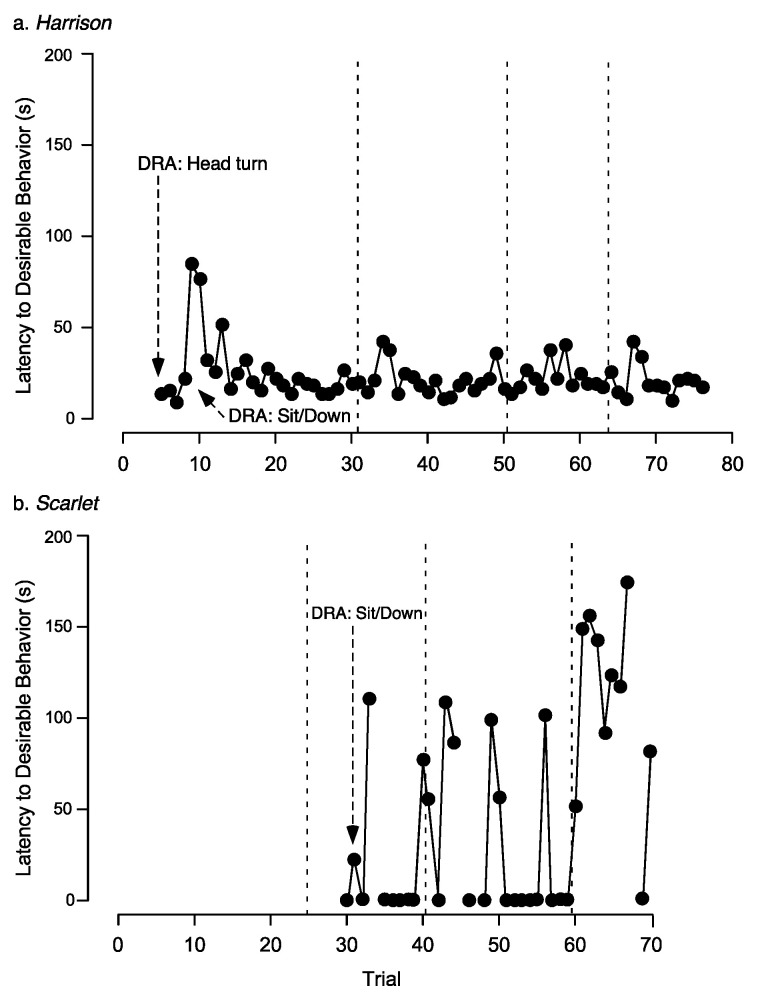
Latency to target desirable behaviors across trials for the two dogs (**a.** Harrison; **b.** Scarlet) in the DRA condition. The trials on which the various DRA schedules were first started are indicated by arrows. Dotted vertical lines indicate the end of one session and the start of a new session. Latencies that are 0 indicate that the dog was engaged in the desirable behavior (e.g., lying down) as the owner left and did not get up when the owner left. Instead, it remained in that position and, after the specified interval of required good behavior elapsed, met the contingency for owner return.

While we did not address this question directly in our current study, we did measure Harrison’s and Scarlet’s latency to engage in the desirable behaviors specified by the DRA across trials (Figure 4) and this could potentially inform our use of DRA in this setting. Latencies were variable within and across dogs. For both Harrison and Scarlet, there was no clear evidence that latency to desirable behavior decreased across trials. As per our criteria, we only reinforced the occurrence of the alternative behavior immediately (DRA + 0 s) on two trials before moving on to a DRA + 5 s. Thus, the desirable behavior we identified in the DRA only contacted immediate reinforcement twice before the reinforcer was delayed by at least 5 s. Given the importance of immediate reinforcement to strengthen a response [32], it is possible that if we had more trials in which the target behavior was immediately reinforced, we would have seen a more substantial decrease in the latency to desirable behavior.

In a prior study investigating treatment of SRPB, the initial criteria used was an owner departure time of 5 min [18]. For all of the dogs in our study, the latency to PB was less than 2 min so that this departure time would have continued to elicit PB. Repeatedly subjecting the dog to durations that are long enough to elicit PB and then have the owner return when the PB is occurring or recently occurred can potentially make the PB worse [21], especially considering owner access is a reinforcer [22]. Despite an errorless learning approach [33] in our study, we did still increase criteria too much and the dogs exhibited PB in some trials. Thus, we are able to evaluate the effects of eliciting PB in one trial on subsequent trials.

First, we looked at the trials in which the dog exhibited PB and calculated the percentage of trials subsequent to those first trials with PB that also contained PB (Table 3). On average, 30% of the time that a dog emitted PB on one trial, it would show PB on the subsequent trial and ranged from 0% (Scarlet) to 50% (Tristan). This is despite the criteria being relaxed on that subsequent trial, suggesting that avoiding increasing the criteria too quickly and eliciting PB is advised as it can lead to a backsliding of behavior. 

Second, there were 18 instances in which the dog showed PB on at least two contiguous trials and the owner had exited the room, such that we had latency from owner exit for those trials. In 14 of these instances, the PB occurred sooner after owner departure in the second trial than the first trial in this sequence. This again suggests that once the dog was pushed over the threshold, it became sensitized again to owner departure and supports the conclusion that increasing criteria should be done carefully and with the goal of increasing gradually so as to not elicit PB. 

When PB did occur, we had owners return only after the dog had stopped engaging in the PB and when 5 s without PB had elapsed. We had owners return soon after PB ended to prevent the dog from engaging in further, and potentially more severe, PB. However, the consequence (owner return for 1 min) was the same for these trials as those with no PB. On trials in which PB was exhibited, we hoped the reinforcer would function to reinforce the 5 s absence of PB and not the preceding PB. Additionally, given the temporal delay of the reinforcer from PB, we expected that differential reinforcement of trials with PB might not have had observable effects on the future likelihood of the dog exhibiting PB or not. However, this is a potential limitation of the study as it possible that differential reinforcement of those two trial types (PB or no PB) could have impacted treatment efficacy and progress. It is possible that not differentially reinforcing these trials is one reason we sometimes saw PB on the next trial, despite relaxing the criteria. Evaluating whether differential reinforcement of trials with or without PB increases treatment progress would be a useful future research direction. 

## 4. Discussion 

Our study is the first to report trial-by-trial direct observation measures of a training plan for SRPB with each session implemented under the guidance of the trainer. Typically, instructions for treatment of SRPB are given to the owners who are left to attempt to implement a treatment plan correctly on their own. Treatment outcome is typically measured by owner report, which is prone to erroneously reporting success even when there is not any, including being more likely to report improvement when they have invested effort and resources into the treatment [17], with no assessment of treatment integrity or direct observation of the dog’s behavior. A few studies have improved on this typical data collection approach. In one, owners record perceived SRPB after each departure, rather than recalling the dog’s behavior over the past week or weeks [18], which necessarily contaminates the data with the influence of memory [34]. The researchers also augmented owner reports with a few independent observer evaluations as well. In a second study, owner reports were supplemented with actual video footage of the dog being left alone during one absence that the researchers scored [9]. Nevertheless, treatment integrity was not evaluated, and criteria changes were largely left to the owner’s discretion about how quickly to increase owner absence duration. 

Other research has demonstrated that using a remote food delivery apparatus to operantly reinforce non-barking behavior successfully reduced barking in dogs that vocalized during owner absence [35], and a remote training device dispensing food has been used successfully to reduce symptoms of separation anxiety [36]. Automated approaches could be very useful, given the slow training progress identified in our study and alluded to in others. Nevertheless, for dogs that will not eat in the absence of the owner, our results suggest that owner return could be used effectively as a reinforcer. Owner return is likely a natural reinforcer. The benefit of a natural reinforcer is that, since it regularly occurs (owners return), it can be used easily to maintain desirable behavior without owners having to prepare and deliver anything special (e.g., food) to maintain the behavior. 

In our study, we had owners return and give their dogs attention for 1 min as a reinforcer. This is contrary to many of the behavioral treatments that the pharmacological studies asked owners to implement, in which owners were encouraged to ignore the dog’s greeting behavior, e.g., [14]. The addition of attention might have little effect, and the return of the owner, regardless of attention, could be the potent reinforcer [2]. Ignoring the dog might not be a useful intervention. However, the effects of owner return with and without attention should be evaluated. As noted earlier, the components in many behavioral treatment plans that were provided as adjunctive treatment in the pharmacological studies on SRPB are not evidence-based interventions [13,14] and do not directly address the behavioral issue (e.g., advice such as not allowing the dog to sleep on the bed or in the bedroom). These seem unlikely to solve the behavioral issue as they do not address the behavior directly; at best they could provide some precursor skills for the dog being separated from the owner. However, reports of contextual control over SPRB [1] argues against generalization occurring. Our study also suggests that other recommendations such as having the owners ignore the dog during departure and when they return are likely without merit; we saw within- and across-session improvement in all dogs when the owners gave them a verbal cue that they were leaving and attention when they returned. However, we did not directly address the effects of departures in which the owners completely ignored the dog, compared to departures in which the owner provided calm attention (as in our study) or provided excited attention. It is possible that the type of attention provided upon departure could affect the likelihood of SRPB after departure. Regardless, we expect that reliable, consistent contingencies for the dogs (consistent antecedents that reliably predict consequences for certain behaviors) are likely a critical factor in successful treatment of SRPB. We recommend that treatment advice directly address the behavioral issue (i.e., the dog’s behavior during owner absence) and the contingencies involved, including antecedents and consequences, rather than giving owners more and potentially ineffective tasks. 

The desensitization approach suggests that treatment effect is due to the animal habituating to longer and longer durations of owner absence. Given that owner access is a reinforcer for dogs [22], it is possible that the desensitization protocol suggested actually is based on operant contingencies that have not been made explicit; that is, the treatment effects observed have been due to owner return reinforcing desirable behavior rather than the dog habituation to owner absence. We hope future research can more explicitly explore this possibility. 

Interestingly, a prior study in which the treatment approach was described as desensitization also described their procedure as utilizing “the owner’s return which culminates in an extraordinary reward period for the dog” [1] (p. 244), which rings of operant contingencies (as did their use of punishment when the dog engaged in SRPB). Even more explicitly, they described establishing discriminative stimuli for appropriate behavior utilizing differential reinforcement. They seemed aware that owner return could function as an important reinforcer for this behavioral issue, despite not explicitly stating operant contingencies. 

If the desensitization protocol is simply an imprecise operant treatment plan that does not make the response contingency explicit, it might not be as effective as an operant protocol that does make the contingencies explicit. For example, in a desensitization plan (in which owner return is not response-contingent) the owner could inadvertently return while the dog is engaged in PB, thereby reinforcing PB and slowing treatment progress. Future research should compare the effectiveness of an operant approach to treating SRPB and desensitization to determine whether either is more effective, and if either is easier to implement by the average owner. It is possible that an operant approach is more successful as a treatment approach but, as it requires precise timing, observation of behavior, understanding of contingencies, and how to increase criteria across trials, much too hard and complex for an owner to implement. As such, a noncontingent desensitization approach, while less effective or efficient, might be the preferred treatment if an owner is to implement the treatment without guidance from a professional, especially given the evidence that owner compliance is low, especially with more challenging intervention components [4]. Nevertheless, even taking a desensitization approach, “most owners need individual assistance to implement the treatment plan” [1] (p. 253). 

The challenges of owners implementing treatment plans with guidance from professionals but without monitoring of treatment integrity or help with criteria changes is evidenced by the fact that seven out of eight dogs still showed some SRPB in at least one of the last five owner departures [18], of 42 owners contacted after clinical treatment for SRPB who still owned their dogs, only 46% thought their dogs improved, and after 6 or 12 weeks of behavioral treatment, with check-ins every 3 weeks, between 15% and 20% of owners (at 6 weeks) and 10–25% of owners (at 12 weeks), depending on the order of treatment component delivery, reported that their dogs had either shown no improvement or worsened [9]. Additionally, while these dogs did decrease the time spent engaging in PB, after 12 weeks of treatment, they still engaged in a mean of 25 s of PB during the first hour of owner absence. Given the severity of this behavioral issue, any instance of PB could still be a significant issue for the owner. 

## 5. Conclusions

Our data demonstrate within- and across-session progress for all dogs, indicating that contingent owner return can be an effective treatment for SRPB. While the progress was generally slow, some dogs were especially slow in their response to this treatment. Nevertheless, the progress we made is not unlike that reported in other studies. Using a remote food delivery apparatus to reinforce the absence of barking in dogs whose owners reported problematic barking when the owners were gone, one dog received over 7 hrs of treatment to be at the level where the dog could be silent for 10 min in order to receive food, a second received 4 hrs of treatment and could be silent for 6 min, and a third dog received over 3 hrs of treatment and could be silent for 20 min [35]. Of course, part of the number of sessions at each criterion was to allow the researchers to clearly demonstrate experimental control, but treatment is still likely slow. A more recent study using an automated food delivery system required over 9 h of training for a dog diagnosed with separation anxiety to be able to stay alone for 30 min [36], although this did not require the owner to be present and active during all 9 hrs of training, which makes this treatment likely more attractive for owners. Still, it demonstrates that treatment for SRPB is typically slow; practitioners set expectations of weeks to months for treatment [37]. While we did not reach durations of owner absence that would allow owners to go to work or run errands, it is possible that with continued training the dogs could continue to increase the time they could successfully stay by themselves. Nevertheless, given how slowly our treatment proceeded, more research is warranted into how to make treatment most effective, efficient, and practical for owners to implement, including adding Pavlovian counterconditioning to address the negative valence of owner departures for dogs and employing remote training devices as much as possible.

## Figures and Tables

**Table 1:** Dog Demographics. Breeds are based on owner reports. Ages are reported in months (m) and years (y). Sex is indicated by M (male) or F (female). N indicates the dog is neutered and S indicates the dog is spayed.

Dog	Breed	Age	Sex
Harrison	Rhodesian ridgeback x	2 y	NM
Mei	Boston terrier	8 m	SF
Sadie	Terrier x	3 y	SF
Scarlet	Pit bull/Catahoula x	2 y	SF
Tristan	Chihuahua	3 y	NM

**Table 2:** Behavioral Definitions. Definitions follow those of prior research [20]. Behaviors were coded as duration (D) or frequency (F).

Code	Behavior	Behavioral Definition
EX	Exploration (D)	Motor activity directed toward physical aspects of the environment, including sniffing, and gentle oral examination, such as licking
LO	Locomotion (D)	Walking or running around without exploring the environment
OB	Oral behavior (D)	Any vigorous behavior directed toward the environment/cage using the mouth (including chewing, biting, shaking, pulling with the mouth)
SC	Scratching (D)	All active behaviors resulting in physical contact with the cage/door, including scratching the cage/door with the paws, jumping on the cage/door, handling with the forelimbs
OE	Oriented to the environment (D)	Sitting, standing, or lying down (the head does not rest on the ground) with obvious orientation toward the physical or social environment, including sniffing, close visual inspection, distant visual inspection (vigilance or scanning)
PA	Passive behavior (D)	Lying down with the head on the ground without any obvious orientation toward the physical or social environment
PT	Panting (D)	Panting
WH	Whining (D)	Whining
BA	Barking (D)	Barking
CI	Circling (D)	Movement of the dog in circles
PL	Play (D)	Any vigorous or galloping gaited behavior directed towards a toy; including chewing, biting, shaking from side to side, scratching or batting with the paw, chasing rolling balls, and tossing using the mouth. Although the dog may take the objects into its mouth, destruction is not included in this category
GR	Grooming (D)	Action of cleaning the body surface by licking, nibbling, picking, rubbing, scratching, et cetera, directed toward the animal’s body
‘	Ears back (D)	Ears flattened and back
HO	Howling (D)	Howling
TR	Trembling (D)	Trembling/shaking movements of the body or head
PU	Paw up (D)	Front limb raised
LL	Lip licking (F)	Part of tongue is shown and moved along the upper lip
YA	Yawning (F)	Yawning
EL	Elimination (F)	Defecation or urination in sitting or standing position

**Table 3:** Descriptive statistics of baseline and treatment. Longest time alone without problem behavior (PB) exceeded highest criteria completed as it took owners a second or more to return once the contingency was met and dogs in differential reinforcement of alternative behavior (DRA) did not always immediately emit the target behavior immediately. Conditional probability is the probability that a dog would exhibit PB on a subsequent trial if it had exhibited PB on the previous trial; this is despite the subsequent trial having lower criteria than the first trial when the dog exhibited PB.

Dog	Latency to PB (Baseline)	Number of Treatment Trials	Highest Criteria Completed	Longest Time Alone without PB	Percentage Successful Trials	Conditional Probability of PB
Harrison	34.8 s	76	300 s DRA (lying down)	326.8 s	96.1	0.33
Mei	1.2 s	65	120 s DRO	122.2 s	67.7	0.38
Sadie	7.6 s	148	60 s DRO	64.0 s	75.0	0.30
Scarlet	96 s	70	150 s DRA (lying down)	247.7 s	94.3	0.00
Tristan	0 s	90	10 s DRO	12.3 s	64.4	0.50
Mean	27.1 s	89.8	128 s	154.6 s	79.5	0.30

**Table 4:** Target behaviors. Problem behaviors identified from Baseline for each dog and other behaviors that were not problem behaviors but were undesirable and would not be reinforced during differential reinforcement of other behavior (DRO). Specific desirable behaviors reinforced in a DRA for Harrison and Sadie.

Dog	Problem Behaviors (Baseline)	Additional Behaviors Not Reinforced (DRO)	Specific Desirable Behaviors Reinforced (DRA) *(if Applicable)*
Harrison	Whining, scratching, oral behavior (destruction)	N/A	Head turn (started Trial 4); Sit/down (started Trial 8)
Mei	Barking, whining, scratching	N/A	N/A
Sadie	Barking, whining	N/A	N/A
Scarlet	Oral behavior (destruction)	Vigilance (running to window)	Sit/down (started Trial 31)
Tristan	Barking	N/A	N/A

**Table 5:** Behavioral criteria during treatment. Criteria changes for dogs in the DRO or DRA. Moving to the next criteria required two successful trials without problem behavior at the previous level. If problem behavior occurred on one trial, the criteria returned to the last successful criteria. For Sadie and Tristan, initial DRO criteria varied until the owner could successfully exit the house without the dog exhibiting PB, after which they followed the DRO schedule.

DRO	DRA
Owner exit + immediate return	Target behavior + immediate return
Owner exit + 5 s	Target behavior + 5 s
Owner exit + 10 s	Target behavior + 10 s
Owner exit + 15 s	Target behavior + 15 s
Owner exit + 30 s	Target behavior + 30 s
Owner exit + 45 s	Target behavior + 45 s
Owner exit + 60 s	Target behavior + 60 s
Owner exit + 90 s	Target behavior + 90 s
Owner exit + 120 s	Target behavior + 120 s
Owner exit + 150 s	Target behavior + 150 s
Owner exit + 180 s	Target behavior + 180 s
Owner exit + 210 s	Target behavior + 210 s

**Table 6:** Criteria changes for dogs across sessions. The sessions (1–4) for each dog are shown. The number of trials in that session is given in (). Initial Target Criteria reflect the treatment goal for Trial 1 of that session. Initial Successful Criteria reflect the criteria in place when the dog had its first successful trial in that session (no PB). End Successful Criteria reflect the last criteria in place when the dog has its last successful trial in that session. Highest Successful Criteria reflect the highest criteria in place during that session in which the dog had a successful trial.

Dog *Session*	Initial Target Criteria	Initial Successful Criteria	End Successful Criteria	Highest Successful Criteria
**Harrison**				
1 (30)	DRO + 0 s	DRO + 0 s	DRA (sit/down) + 30 s	DRA (sit/down) + 30 s
2 (20)	DRA (sit/down) + 10 s	DRA (sit/down) + 10 s	DRA (sit/down) + 60 s	DRA (sit/down) + 60 s
3 (14)	DRA (sit/down) + 30 s	DRA (sit/down) + 30 s	DRA (sit/down) + 210 s	DRA (sit/down) + 210 s
4 (12)	DRA (sit/down) + 30 s	DRA (sit/down) + 30 s	DRA (sit/down) + 300 s	DRA (sit/down) + 300 s
**Mei**				
1 (13)	DRO + 0 s	DRO + 0 s	DRO + 5 s	DRO + 5 s
2 (18)	DRO + 0 s	DRO + 0 s	DRO + 5 s	DRO + 5 s
3 (11)	DRO + 30 s	DRO + 0 s	DRO + 15 s	DRO + 15 s
4 (23)	DRO + 10 s	DRO + 10 s	DRO + 30 s	DRO + 120 s
**Sadie**				
1 (32)	DRO + 0 s	DRO + 0 s	DRO + 0 s	DRO + 10 s
2 (44)	DRO + 0 s	Owner steps over barrier + 5 s	DRO + 5 s	DRO + 15 s
3 (33)	DRO + 0 s	Owner steps over barrier + 0 s	DRO + 60 s	DRO + 60 s
4 (39)	DRO + 0 s	Owner steps over barrier + 0 s	DRO + 10 s	DRO + 15 s
**Scarlet**				
1 (25)	DRO + 0 s	DRO + 0 s	DRO + 150 s	DRO + 150 s
2 (15)	DRO + 15 s	DRO + 15 s	DRA (sit/down) + 90 s	DRA (sit/down) + 90 s
3 (19)	DRA (sit/down) + 30 s	DRA (sit/down) + 30 s	DRA (sit/down) + 120 s	DRA (sit/down) + 120 s
4 (11)	DRA (sit/down) + 60 s	DRA (sit/down) + 60 s	DRA (sit/down) + 120 s	DRA (sit/down) + 150 s
**Tristan**				
1 (14)	DRO + 0 s	Walks past crate + 0 s	Opens door halfway + 0 s	Opens door fully + 0 s
2 (18)	Opens door fully + 0 s	Opens door fully + 0 s	Opens door halfway + 0 s	Opens door fully + 0 s
3 (33)	Foot across threshold + 0 s	Step to threshold + 0 s	DRO + 0 s	DRO + 0 s
4 (25)	Foot across threshold + 0 s	Step to threshold + 0 s	DRO + 10 s	DRO + 10 s
